# Prevalence and Associated Factors of Thinness among Adolescent Students in Finote Selam Town, Northwest Ethiopia

**DOI:** 10.1155/2020/9170301

**Published:** 2020-06-02

**Authors:** Damitie Kebede Mengesha, Reddy P. C. J. Prasad, Degnet Teferi Asres

**Affiliations:** ^1^Faculty of Chemical and Food Engineering, Bahir Dar Institute of Technology, Bahir Dar University, P. O. Box 79, Bahir Dar, Ethiopia; ^2^College of Agriculture and Environmental Sciences, Bahir Dar University, P. O. Box 5501, Bahir Dar, Ethiopia

## Abstract

Even if adolescence is a window of opportunity to break the intergenerational cycle of malnutrition, adolescents are the neglected age groups. Hence, information regarding the nutritional status of adolescents is lacking, making creating and implementing intervention programs difficult. This study aimed to assess the prevalence and determinants of thinness among school adolescents in Finote Selam Town, Northwest Ethiopia. A school-based cross-sectional study among adolescent students aged 10–19 in public primary and secondary schools was carried out in Finote Selam Town from February 05 to March 27, 2018. Stratified and simple random sampling techniques were employed to select study subjects. A total of 397 adolescent students were included in the study. Pretested structured questionnaires were used to collect the data. Data were entered using Epi Info version 7 and analyzed using SPSS version 20 and WHO AnthroPlus software. A multivariable binary logistic regression analysis was employed to identify factors associated with thinness. Crude and adjusted odds ratios with 95% level significance were used to measure the strength of association, and statistical significance was declared at *p* value less than 0.05. The prevalence of thinness among adolescents was 14.9%. Early adolescent stage (AOR = 4.81; 95% CI : 1.23, 18.51), being male adolescent students (AOR = 2.33; 95% CI : 1.60, 3.40), having less than 1000 birr family monthly income (AOR = 6.54; 95% CI : 3 : 82, 14.89), having 1000–2000 birr family monthly income (AOR = 3.47; 95% CI : 1.15, 7.45), and using well water (AOR = 3.82; 95% CI : 1.46, 10.04) were significantly associated with thinness at 95% confidence interval. The study revealed that prevalence of thinness was high in the study area. Sex, place of residence, and family monthly income were found to be important factors associated with thinness among the respondents.

## 1. Introduction

Both the UN and WHO define adolescence as a segment of population group age from 10 to 19 years old; it is a transition period from childhood to adulthood and has intense physical, psychosocial, and cognitive development [1]. During this period, the final growth spurt occurs; particularly, early adolescence after the first year of life is the critical period of rapid physical growth and changes in body composition, physiology, and endocrine [2]. Up to 45% of skeletal growth takes place, and 15 to 25% of adult height is achieved during adolescence; during the growth spurt of adolescence, up to 37% of total bone mass may be accumulated [3]. Regarding body composition change, girls begin to store fat around the breast, hips, and upper arm but boys start losing fat and develop muscle [4].

The adolescence period is a window of opportunity for human beings because there is possibility of little catch-up growth [5], and it is a time of changing lifestyles and food habit; changes affect both nutrient needs and intake, so it is an opportunity to shape this new behavior adoption [6]. Our world is a home to 1.8 billion (24.66%) young people between the ages of 10 and 24, and the youth population is growing fastest in the poorest nations; currently, adolescents make up roughly 20% of the global population [7]. In developing countries, adolescents have an even higher 85% demographic weight, for instance, roughly 26% in Salvador, compared to 14% in the USA [8]. Similarly, in Ethiopia, children and adolescents constitute about 48% of Ethiopian population, and about 25% of the Ethiopian populations are adolescents, but studies among this age group were insufficient [9].

Different researchers conducted a study on thinness among school adolescents in different parts of the country. The prevalence of thinness varies across different regions of Ethiopia. The prevalence of thinness among adolescent girls was 21.6%, 28%, 21.4%, and 14.8% in studies conducted in Babille District [10], Bedlle [11], Adwa [12], and Arsi Zone [13]. The overall prevalence of thinness among adolescent school girls in Lay Guyint Woreda, Northwest Ethiopia, was 29.0% [14]. The prevalence of thinness among adolescent school girls in Adwa Town, North Ethiopia, was 21.4% [12]. The overall prevalence of thinness among school-going adolescents in Mekelle City, Northern Ethiopia, was 37.8% [15]. The prevalence of thinness among adolescents in Wukro, Northern Ethiopia, was 26.1% [16]. However, there was no any study report on thinness in the study area. This study would serve as a baseline for further study and be important for designing intervention and a guide for policymakers and development planners. This study was mainly focused to assess the prevalence and explore determinants of thinness among school adolescents in Finote Selam Town, Northwest Ethiopia.

## 2. Methods

### 2.1. Study Design and Area

The study design was a school-based, cross-sectional study among adolescent students aged 10–19 in public primary and secondary schools. The study was carried out from February 05 to March 27, 2018. This study was conducted in Finote Selam Town which is located 389 km northwest of Addis Ababa and 176 km southeast from Bahir Dar, the city of Amhara Region. Finote Selam town administration has been established to be the main town of West Gojjam Zone since 2004 E.C. The total population size of the town according to 2007 census report was 25913 (13, 035 males and 12, 878 females). The town is situated at an altitude ranging from 1500–2300 meters above sea level and has an area coverage of 116,954 ha. The area receives an average annual rainfall of 1250 mm. The minimum and maximum daily temperatures of the area are 14 and 32°C, respectively. It is bordered on the north by Sekela and Quarit, on the south by Dega Damot and Burie, on the east by Deg damot and Dembecha, and on the west by Burie. There are six primary schools, one high school, one preparatory school, and five colleges in Finote Selam Town. The total number of students from grade 5–12 was 12,323 [17].

### 2.2. Source and Study Population

All adolescent students (10–19) in Finote Selam town schools were the source population. Randomly selected adolescent students (10–19) in Finote Selam town schools were study population.

### 2.3. Sample Size and Sampling Procedure

The minimum sample size required was calculated using single proportion formula. The proportion of thinness among school-going adolescents of Mekelle City, Northern Ethiopia, was 37.8% [18] at a 95% confidence level, 5% margin of error adding 10% as contingency for nonresponse rate:

(1) $$\begin{array}{c} n={\left(\frac{z}{d}\right)}^{2}xP\left(1-p\right), \end{array}$$

where *n* = sample size, *Z* = *Z* score at 95% CI = 1.96, *p* = 37.8%, and *d* = marginal error = 105:

(2) $$\begin{array}{c} n=\frac{{\left(1.96\right)}^{2}*0.378\left(1-0.378\right)}{{\left(0.05\right)}^{2}},\\ =\frac{3.8416*0.378*0.622}{{\left(0.05\right)}^{2}},\\ =361. \end{array}$$

By adding 10% nonresponse rate, the minimum sample size required to estimate the prevalence of thinness and its associated factors among adolescent students was 361 + 10% (361 + 36) = 397.

**Figure 1:**
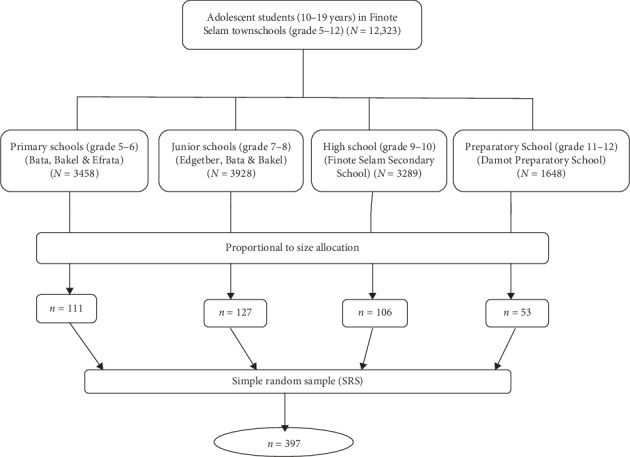
Schematic presentation of sampling procedures.

To obtain the sample, stratified random sampling technique was used. The schools were stratified into primary schools, junior schools, high school, and preparatory school. Three primary schools, namely, Bata, Bakel, and Efrata, and three junior schools, namely, Edgetber, Bata, and Bakel, were selected using simple random sampling from six primary schools and six junior schools, respectively, whereas a high school, namely, Finote Selam Secondary School, and a preparatory school, namely, Damot Preparatory School, were selected purposively since one high school and one preparatory school were present in Finote Selam Town. The total sample size was distributed proportionally to the schools. The sampling frame was students' identification number in their respective school. Numbers of students to be included in the study were determined by a simple random sampling method (Figure 1).

### 2.4. Data Collection

To generate the data set used in this study, pretested structured questionnaires were used to collect data by trained data collectors. Explanatory variables were selected after conducting a detailed literature review from related articles to collect data on the sociodemographic, nutritional, and health-related variables [16–19]. The questionnaires were translated into the local language (Amharic) for easy understanding by the respondents. Each student was interviewed to obtain information on sociodemographic, nutritional, and health-related characteristics of the adolescents' family.

Anthropometric data were collected by trained data collectors who were health extension workers. And the overall activity was coordinated by the investigator. The age of the adolescents was derived from the school register. Height and weight were measured using a stadiometer and Seca Digital Scale (Seca, Germany), respectively. The weight was recorded to the nearest 0.1 kg. It was calibrated against the known weight regularly. Before the real anthropometric data collection, a standardization exercise was performed during the training to capture technical error of measurement. During the procedure, the subjects wore light clothes and took off their shoes. Height was measured in cm using a portable stadiometer. All adolescents were measured against the wall without foot wear and with heels together and their heads position and eyes looking straight ahead (Frankfurt plane) so that the line of sight was perpendicular to the body. The height was recorded to the nearest 0.1 cm. The same measurer was employed for a given anthropometric measurement to avoid variability.

### 2.5. Data Quality Control and Management

To ensure the reliability and validity of the study, training was given for the data collectors, the data collection was done by two health extension workers, and close follow-up was done by the investigator during data collection. The Amharic version of the questionnaire was tested on 5% of the samples at the Selamamba Primary School, who have similar characteristics with the study participant but did not participate in the study. The data collectors and investigator were participated on pretesting and standardization of the questionnaires. Problems highlighted during the preliminary study were corrected before the start of the actual survey. Each question was properly coded; continuous supervision was performed during the pretest and data collection period by the investigator. Completeness and consistency of recording on the questionnaire sheets were evaluated by the investigator at the end of each working day so that correction measures were taken for the next time.

### 2.6. Data Analyses

Sociodemographic, anthropometric, nutrition, and health-related data were entered into Epi Info version 7 and checked for completeness and consistency, followed by data cleaning and editing on Epi Info. Then, the data were analyzed by using SPSS (Statistical Package for Social Sciences), version 20 software. WHO AnthroPlus software was used for assessing growth of the adolescents [20]. Descriptive statistics using frequencies and proportions was used to present the study results. For anthropometric data analysis, if the BMI-for-age *Z*-score is below minus two standard deviations (−2 SDs) from the median of the reference population, then the child is thin. Odds ratio with 95% confidence interval was used for checking the strength of associations between the outcome variable (thinness) and independent variables. Bivariate logistic regression was performed, and the variable with *p* value less than 0.25 was transported into multivariable binary logistic regression analysis to identify the determinant of malnutrition of under-five children. Finally, variables with *p* values <0.05 in the multivariable logistic regression model were taken as statistically significant [21].

## 3. Results

### 3.1. Sociodemographic Characteristics of Study Participants and Their Family

From a total of 397 adolescent students who were selected as a sample, with 100% response rate, all study participants were involved in this study. Among these, 47 (11.8%) were early adolescents, 151 (38.0%) were midadolescents, and 199 (50.1%) were late adolescents. From the participants, 108 (27.2%) were from primary schools, 132 (32.2%) were from junior schools, while 116 (29.2%) were from the high school, and others 41 (10.3%) were attending the preparatory school. Of the total participants, 195 (49.1%) were from urban areas, whereas 202 (50.9%) were from rural areas (Table 1).

### 3.2. Nutrition and Health-Related Characteristics of School Adolescents

Most of the adolescents, 278 (70.0%), were consuming meals three and above times per day, whereas 119 (30.0%) of adolescents were consuming meals two times per day. Among the respondents, 33.0% were reported having illness in the last one month. Among the respondents, 288 (72.5%) had home gardens. Vegetables (46.1%) and fruits (51.4%) were consumed daily (Table 2).

### 3.3. Anthropometric Results

The minimum and maximum heights of study subjects were 127.50 cm and 186.70 cm, respectively. The mean ± SD overall height of the participants was 158 ± 10.67 cm. Similarly, the minimum and maximum weights of study participants were 20.5 kg and 68 kg, respectively. The mean ± SD overall weight of the participants was 45.99 ± 10.09 kg. The mean heights of boys and girls were 158.99 ± 12.24 cm and 156.31 ± 6.94 cm, respectively. Similarly, the mean weights of boy and girl adolescents were 45.1 ± 10.97 kg and 47.53 ± 8.17 kg, respectively. The mean age of the study participants was 15.54 years (15.54 ± 2.41 SD).

*Prevalence of thinness*: the overall prevalence of thinness among adolescent students at Finote Selam Town was 14.9%.

**Figure 2:**
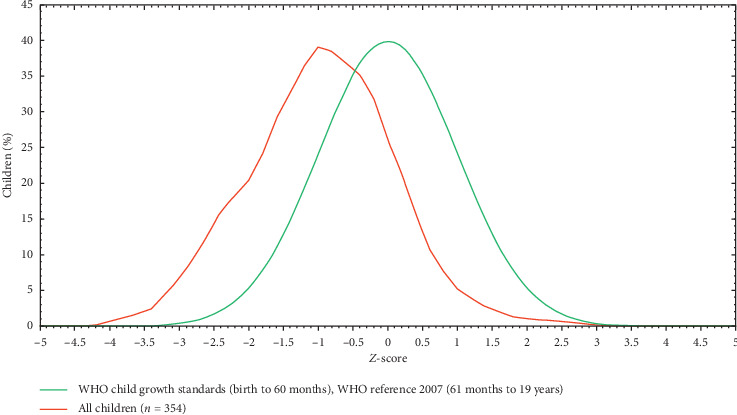
BMI-for-age *Z* -score of adolescent students in Finote Selam Town, Ethiopia.

The mean *Z*-score of BMI-for-age of all adolescents was −1.13 which revealed the distribution of BAZ (Figure 2).

**Figure 3:**
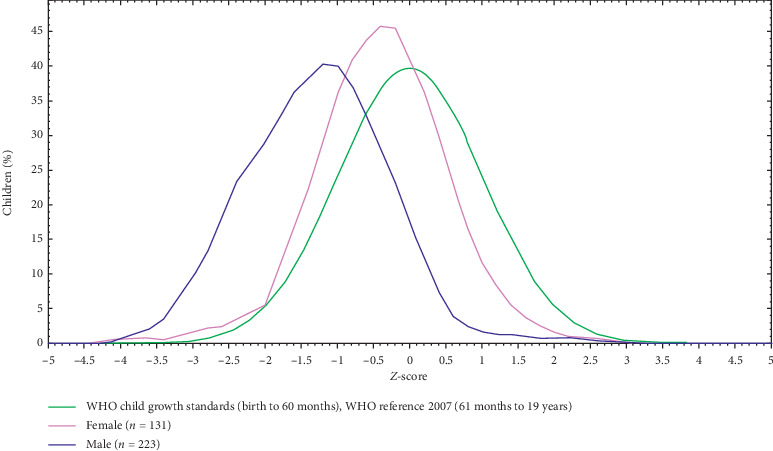
BMI-for-age *Z* -score of by sex of adolescent students in Finote Selam Town, Ethiopia.

The mean *Z*-scores of BMI-for-age among boys and girls were −1.29 and −0.51 that showed the distribution of BAZ, respectively (Figure 3).

### 3.4. Factors Associated with Thinness

Exploring determinants associated with thinness is important to take nutritional intervention action in the study area. Results of the multivariable binary logistic regression model showed that the age, sex, family monthly income, and sources of drinking water were significantly associated with thinness. The odds of thinness were 4.81 times higher among adolescent students in the early adolescent stage as compared to adolescent students in the late adolescent stage (AOR = 4.81; 95% CI : 1.23, 18.51). Male adolescent students had 2.13 times higher odds of thinness (AOR = 2.13; 95% CI : 1.60, 3.40) compared to female adolescent students. The students who came from rural area were 2.16 (AOR = 2.61; 95% CI : 0.96, 4.87) times more likely to be thin compare to those who came from urban area. The students from less than 1000 and 1000–2000 birr family monthly income were 6.54 (AOR = 6.54; 95% CI : 3.82, 14.89) and 3.47 (AOR = 3.47; 95% CI : 1.15, 7.45) times more likely to be thin compared to greater than 2000 birr family monthly income, respectively. Students from households that used well water supply as main source of water supply were 3.82 times more likely to be at risk of being thin than students from households that used tap water supply for human consumption (AOR = 3.82; 95% CI : 1.46, 10.04) (Table 3).

## 4. Discussion

In this study, the prevalence of thinness and associated factors in Finote Selam Town, Northwest Ethiopia, was assessed. The prevalence of thinness was 14.9%, and this finding was lower than the studies in Mekelle City (37.8%) [15], Ambo City (27.5%) [22], Wukro District (26.1%) [16], and Seychelles (27.7%) [23]. Another study in Ethiopia, in Jimma Zone, has reported a much higher level (80.8%) of thinness prevalence among adolescents [24]. The prevalence of thinness of this study was higher than studies done in Addis Ababa (6.2%) (13%) [25]. This divergence might be due to the difference in socioeconomic background, dietary habit, and type of meals among the cities. Findings in other African countries include Burkina Faso (13.7%) [26], Asembo and Mumias, Kenya (15.6%) [27], Tunisia (13%) [28], and Tamale Metropolis district, Ghana (10%) [29]. The variation might be due to socioeconomic background, geographical characteristics of study area, cultural difference in dietary habit, and care practices.

The odds of thinness were 4.81 times higher among adolescent students in the early adolescent stage as compared to adolescent students in the late adolescent stage. This finding was in agreement with findings from Adwa Town, Northen Ethiopia, [17] and in Community-Based Nutrition implementing districts, Amhara Region, Ethiopia [30]. This might be due to as age increases, adolescents might access food easily by themselves; on the other hand, as age increases, adolescents become more matured. Male adolescent students had 2.13 times higher odds of thinness compared to female adolescent students. This study was in line with the studies conducted in Mekelle City, Northern Ethiopia [15], Jimma Zone, Southwest Ethiopia [24], and Wukro, Northern Ethiopia [16] which confirmed that males were more affected in thinness than girls. This might be due to variation of maturation time in boys and girls, for which girls reached maturation earlier than boys.

The students who came from rural area were 2.16 times more likely to be thin compared to those who came from urban area. This finding is in agreement with other previously conducted studies [12, 16]. The variation might be due to food preference, food consumption pattern, and inequalities in dietary diversity between urban and rural areas. The risk of being thin among school adolescents whose mothers did not attend education was 1.78 times more compared to school adolescents whose mother attended college and above education. This finding is consistent with the study that conducted in Adama City, Central Ethiopia [31]. The risk of being thin among school adolescents whose mothers attended primary school (1–8) and secondary school (9–12) education was 1.29 and 1.15 (AOR = 1.15; 95% CI: 0.66, 1.97) times more compared to students whose mothers attended college and above education, respectively. This finding is in line with the study that conducted in Adama City, Central Ethiopia [32]. This is due to the fact that if the level of education of the mother is low, her decision-making and her contribution to the total family income will be low. This places the family at risk of not meeting their needs including nutritional needs.

The odds of being thin were 1.90 times higher among school adolescents who lived in household members of ≥5 than school adolescents who had lived in household members <5. This finding was in line with the study that conducted in Adwa Town, North Ethiopia [12]. This might be due to sharing of the available food for the large household members, causing inadequate consumption of food, leading to be thin. The students who did not eat vegetables and fruits at least once per day were 1.30 and 1.23 times more likely to be thin compared to those who ate once per day. This finding is in agreement with the study that conducted in Adwa Town, North Ethiopia [12]. This is might be due to the fact that consuming insufficient vegetables and fruits are leading to poor nutritional status.

The students from less than 1000 and 1000–2000 birr family monthly income were 6.54 and 3.47 times more likely to be thinness compared to greater than 2000 birr family monthly income, respectively. This might be due to the better income level of the family would enhance the nutrition status of the adolescents. Students from households that used well water supply as main source of water supply were 3.82 times more likely to be at risk of being thinness than students from households that used tap water supply for human consumption. This finding was in line with findings from Adwa Town, Northen Ethiopia [17]. This might be due to the fact that impure water is a vehicle for intestinal parasites which leads to loss of appetite, leading to poor nutritional status; this might also be due to repeated infection causing depressed immunity and making the severity and duration of disease more severe, contributing to poor nutritional status of the adolescents.

## 5. Conclusions

The prevalence of thinness was high in the study area. Consistent with this result, the mean *Z*-scores of BMI-for-age were higher in boys than girls. Age, sex, family monthly income, and sources of drinking water were significantly associated with thinness among the respondents. Based on the finding, there should be collaboration among health sectors and education sectors of the town to address school adolescents under nutrition problems of the town.

## Figures and Tables

**Table 1:** Sociodemographic-related characteristics of adolescent students at Finote Selam Town, Ethiopia, 2018.

Variables	Frequency	Percentage
*Age group*		
Early adolescent (10–13)	47	11.8
Midadolescent (14–16)	151	38.0
Late adolescent (17–19)	199	50.1

*Sex*		
Female	148	37.3
Male	249	62.7

*Grade*		
Grade 5–6	108	27.2
Grade 7–8	132	33.2
Grade 9–10	116	29.2
Grade 11–12	41	10.3

*Religion*		
Muslim	22	5.5
Orthodox	375	94.5

*Place of residence*		
Urban	195	49.1
Rural	202	50.9

*Father's education*		
Illiterate	95	23.8
Read and write	101	25.4
Primary school (1–8)	41	10.3
Secondary school (9–12)	92	23.2
College and above	68	17.1

*Mother's education*		
Illiterate	102	25.7
Read and write	109	27.5
Primary school (1–8)	34	8.6
Secondary school (9–12)	89	22.4
College and above	63	15.9

*Father's occupation*		
Daily laborer	57	14.4
Farmer	119	30.0
Merchant	85	21.4
Government/nongovernment employee	136	34.3

*Mother's occupation*		
Daily laborer	5	1.3
Housewife	86	21.7
Merchant	119	30.0
Farmer	107	27.0
Government/nongovernment employee	80	20.2

*Family size*		
<5	173	43.6
≥5	224	56.4

*Monthly family income*		
<1000 birr	77	19.3
1000–2000 birr	107	27.0
>2000 birr	213	53.7

*Source of drinking water*		
Spring water	82	20.7
Well water	106	26.7
Public tap water	26	6.5
Tap water	183	46.1

*Presence of functional latrine*		
Yes	257	64.7
No	140	35.3

**Table 2:** Nutrition and health-related characteristics of school adolescents at Finote Selam Town, Ethiopia, 2018.

Variables	Frequency	Percentage
*Number of meals eaten per day*		
Two times	119	30.0
Three times and above	278	70.0

*Illness reported in the last one month*		
Yes	131	33.0
No	266	67.0

*Availability of home garden*		
Yes	288	72.5
No	109	27.5

*Eating vegetables at least once per day*		
Yes	183	46.1
No	214	53.9

*Eating fruits at least once per day*		
Yes	204	51.4
No	193	48.6

*Eating farm animal products at least once per week*		
Yes	115	29.0
No	282	71.0

*Nutrition and health information*		
Yes	47	11.8
No	350	88.2

**Table 3:** Bivariate and multivariable logistic regression of factors associated with thinness among adolescent school students, Finote Selam Town, Ethiopia, 2018.

Variables	Thinness		COR (95% CI)	AOR (95% CI)
	Yes	No		
*Age Group*				
Early adolescent (10–13)	17 (36.2%)	30 (63.8%)	5.14 (2.39, 11.08)^*∗*^	4.81 (1.23, 18.51)^*∗*^
Midadolescent (14–16)	24 (15.9%)	127 (84.1%)	1.90 (0.99, 3.64)	1.68 (0.57, 4.93)
Late adolescent (17–19)	18 (9.0%)	181 (91.0%)	1	1

*Sex*				
Female	4 (2.7%)	144 (97.3%)	1	1
Male	55 (22.1%)	194 (77.9%)	2.68 (1.88, 3.84)	2.13 (1.60, 3.40)^*∗*^

*Grade*				
Grade 5–6	23 (21.3%)	85 (78.7%)	2.57 (0.91, 7.24)	1.77 (0.40, 7.87)
Grade 7–8	22 (16.7%)	110 (83.3%)	2.08 (0.74, 5.86)	1.33 (0.22, 8.19)
Grade 9–10	10 (8.6%)	106 (91.4%)	1.01 (0.32, 3.15)	1.06 (0.20, 5.58)
Grade 11–12	4 (9.8%)	37 (90.2%)	1	1

*Religion*				
Muslim	1 (4.5%)	21 (95.5%)	1	1
Orthodox	58 (15.5%)	317 (84.5%)	3.82 (0.50, 29.18)	3.90 (0.19, 81.94)

*Place of residence*				
Urban	18 (9.2%)	177 (90.8%)	1	1
Rural	41 (20.3%)	161 (79.7%)	2.44 (1.35, 4.40)^*∗*^	2.16 (0.96, 4.87)

*Father's education*				
Illiterate	15 (15.8%)	80 (84.2%)	2.79 (0.92, 8.45)	4.06 (0.23, 71.76)
Read and write	17 (16.8%)	84 (83.2%)	1.97 (0.71, 5.43)	2.73 (0.31, 24, 29)
Primary school (1–8)	10 (24.4%)	31 (75.6%)	1.96 (0.73, 5.32)	2.36 (0.26, 21.41)
Secondary school (9–12)	11 (12.0%)	81 (88.0%)	1.44 (0.51, 4.10)	0.80 (0.5, 12.90)
College and above	6 (8.8%)	62 (91.2%)	1	1

*Mother's education*				
Illiterate	17 (16.7%)	85 (83.3%)	2.38 (0.77, 7.39)	1.78 (0.86, 3.65)
Read and write	16 (14.7%)	93 (85.3%)	1.86 (0.68, 5.07)	1.40 (0.83, 2.34)
Primary school (1–8)	9 (26.5%)	25 (73.5%)	1.50 (0.55, 4.09)	1.29 (0.61, 2.71)
Secondary school (9–12)	11 (12.4%)	78 (87.6%)	1.33 (0.47, 3.81)	1.15 (0.66, 1.97)
College and above	6 (9.5%)	57 (90.5%)	1	1

*Father's occupation*				
Daily laborer	8 (14.0%)	49 (86.0%)	1.20 (0.47, 3.07)	1.1 (0.55, 21)
Farmer	26 (21.8%)	93 (78.2%)	2.27 (1.12, 4.61)^*∗*^	1.55 (0.45, 5.29)
Merchant	11 (12.9%)	74 (87.1%)	1.18 (0.50, 2.75%)	1.52 (0.55, 4.26)
Government/nongovernment employee	14 (10.3%)	122 (89.7%)	1	1

*Mother's occupation*				
Daily laborer	1 (20.0%)	4 (80.0%)	1.86 (0.19, 18.55)	2.10 (0.11, 38.74)
Farmer	21 (24.4%)	65 (75.6%)	2.74 (1.16, 6.46)^*∗*^	2.16 (0.62, 7.51)
Housewife	15 (12.6%)	104 (87.4%)	1.33 (0.55, 3.23)	2.46 (0.58, 10.47)
Merchant	13 (12.1%)	94 (87.9%)	1.24 (0.51, 3.04)	1.52 (0.43, 5.36)
Government/nongovernment employee	9 (11.2%)	71 (88.8%)	1	1

*Family size*				
<5	17 (9.8%)	156 (90.2%)	1	1
≥5	42 (18.8%)	182 (81.2%)	2.43 (1.32, 4.45)^*∗*^	1.90 (0.80, 4.51)

*Family monthly income*				
<1000	29 (37.7%)	48 (62.3%)	6.10 (3.09, 12.04)^*∗*^	11.54 (3.82, 34.89)^*∗*^
1000–2000	14 (13.1%)	93 (86.9%)	1.71 (0.80, 3.64)	3.47 (1.15, 10.45)^*∗*^
>2000	16 (7.5%)	197 (92.5%)	1	1

*Source of drinking water*				
Well water	39 (21.3%)	144 (78.7%)	4.53 (1.71, 11.98)	3.82 (1.46, 10. 04)^*∗*^
Spring water	13 (12.3%)	93 (87.7%)	3.37 (0.88, 12.89)	2.56 (0.87, 7.56)
Public tap water	2 (7.7%)	24 (92.3%)	1.88 (0.23, 15.49)	1.46 (0.26, 8.09)
Tap water	5 (6.1%)	77 (93.9%)	1	1

*Presence of functional latrine*				
Yes	38 (14.8%)	219 (85.2%)	1	1
No	21 (15.0%)	119 (85.5%)	1.08 (0.49, 2.38)	1.05 (0.59, 1.87)

*Number of meals eaten per day*				
Two times	19 (16.0%)	100 (84.0%)	1.17 (0.64, 2.13)	1.01 (0.53, 1.91)
Three times and above	40 (14.4%)	238 (85.6%)	1	1

*Illness reported in the last one month*				
Yes	19 (14.5%)	112 (85.5%)	1.06 (0.57, 1.96)	1.03 (0.57, 1.87)
No	40 (15.0%)	226 (85.0%)	1	1

*Availability of home garden*				
Yes	50 (17.4%)	238 (82.6%)	1	1
No	9 (8.3%)	100 (91.7%)	1.60 (1.01, 2.54)	1.15 (0.66, 1.99)

*Eating vegetables at least once per day*				
Yes	23 (12.6%)	160 (87.4%)	1	1
No	36 (16.8%)	178 (83.2%)	1.35 (0.76, 2.60)	1.30 (0.72, 2.36)

*Eating fruits at least once per day*				
Yes	26 (12.7%)	178 (87.3%)	1	1
No	33 (17.1%)	160 (82.9%)	1.33 (0.76, 2.31)	1.23 (0.69, 2.18)

*Eating farm animal products at least once per week*				
Yes	14 (12.2%)	101 (87.8%)	1	1
No	45 (16.0%)	237 (84.0%)	1.20 (0.62, 2.29)	1.19 (0.61, 2.34)

*Nutrition and health information*				
Yes	6 (12.8%)	41 (87.2%)	1	1
No	53 (15.1%)	297 (84.9%)	1.15 (0.49, 2.71)	1.08 (0.43, 2.69)

## Data Availability

All data in this study are included in the figures, tables, and statements.

## Abbreviations

AOR: Adjusted odds ratio
CM: Centimeter
COR: Crude odds ratio
CSA: Central statistics agency
CV: Confidence interval
EDHS: Ethiopian demography and health survey
SD: Standard deviation
SPSS: Statistical Package for Social Sciences
UN: United Nations
WHO: World Health Organization.

## References

[1] WHO (2013). *Adolescent Nutrition a Neglected Dimension*.

[2] Namrata C., Archana C. (2013). A comparative study of nutritional intake in adolescentgirls.

[3] Khan M. R., Ahmed F. (2005). Physical Status, nutrient intake and dietary pattern of adolescent female factory workers in Urban Bangladesh. *Asia Pacific Journal of Clinical Nutrition*.

[4] Sally S. (2013). California state, 4-H center for youth development, the biology of adolescence.

[5] UNICEF (2011). *Adolescence an Age of Opportunity the State of the World’s Children*.

[6] Brown J. E., Stang J. (2011). *Nutrition through Life the Cycle*.

[7] Gupta M. D., Engelman R., Levy J., Luchsinger G., Merrick T., Rosen J. E. (2014). *The Power of 1.8 Million Adolescent, Youth and the Transformation of the Future*.

[8] Burt M. R. Why should we invest in adolescents?.

[9] CSA, Centeral Statistics Agency of Ethiopia, Addis Ababa, 2007

[10] Teji K., Dessie Y., Assebe T. (2016). Anaemia and nutritional status of adolescent girls in Babile district, Eastern Ethiopia. *Pan African Medical Journal*.

[11] Wolde T., Mekonnin W. A. D., Yitayin F. (2014). Nutritional status of adolescent girls living in southwest of Ethiopia. *Food Science and Human Wellness*.

[12] Gebregyorgis T., Tadesse T., Atenafu A. (2016). Prevalence of thinness and stunting and associated factors among adolescent schoolgirls in Adwa Town, North Ethiopia. *International Journal of Food Science*.

[13] Yemaneh Y., Girma A., Nigussie W. (2017). Undernutrition and its associated factors among adolescent girls in the rural community of Aseko district, Eastern Arsi Zone, Oromia region, Eastern Ethiopia, 2017. *International Journal of Clinical Obstetrics and Gynaecology*.

[14] Arage G., Assefa M., Worku T. (2019). Socio-demographic and economic factors are associated with nutritional status of adolescent school girls in Lay Guyint Woreda, Northwest Ethiopia. *SAGE Open Medicine*.

[15] Gebremariam H., Omer S., Assefa H. (2015). Assessment of nutritional status and associated factors among school going adolescents of Mekelle City, Northern Ethiopia. *International Journal of Nutrition and Food Sciences*.

[16] Melaku Y. A., Zello G. A., Gill T. K., Adams R. J., Shi Z. (2015). Prevalence and factors associated with stunting and thinness among adolescent students in Northern Ethiopia: a comparison to World Health Organization standards. *Archives of Public Health*.

[17] FTEO. Finoteselam Education Office; 2017

[18] Birru S. M., Tariku A., Belew A. K. (2018). Improved dietary diversity of school adolescent girls in the context of urban Northwest Ethiopia. *Italian Journal of Pediatrics*.

[19] Mohammed A. Y., Tefera T. B. (2015). Nutritional status and associated risk factors among adolescents girls in Agarfa High School, Bale Zone, Oromia Region, South East Ethiopia. *International Journal of Nutrition and Food Sciences*.

[20] WHO (2009). *AnthroPlus for Personal Computers Manual: Software for Assessing Growth of the World’s Children and Adolescents*.

[21] Hosmer D. W., Lemeshow S. (2000). *Applied Logistic Regression*.

[22] Alebachew Z. (2010). *Prevalence of Childhood and Adolescent Overweight and Obesity Among Elementary School Students*.

[23] Bovet P., Kizirian N., Madeleine G., Blössner M., Chiolero A. (2011). Prevalence of thinness in children and adolescents in the Seychelles: comparison of two international growth references. *Nutrition Journal*.

[24] Assefa H., Belachew T., Negash L. (2013). Socioeconomic factors associated with underweight and stunting among adolescents of Jimma Zone, South West Ethiopia. *ISRN Public Health*.

[25] Yetubie M. (2012). Anthropometric assessment of adolescent malnutrition in elementary and secondary schools of ambo.

[26] Daboné C., Delisle H. F., Receveur O. (2011). Poor nutritional status of schoolchildren in urban and peri-urban areas of Ouagadougou (Burkina Faso). *Nutrition Journal*.

[27] Leenstra T., Petersen L. T., Kariuki S. K., Oloo A. J., Kager P. A., Kuile F. O. t. (2005). Prevalence and severity of malnutrition and age at menarche; cross-sectional studies in adolescent schoolgirls in western Kenya. *European Journal of Clinical Nutrition*.

[28] Aounallah-Skhiri H., Romdhane H. B., Traissac P. (2008). Nutritional status of Tunisian adolescents: associated gender, environmental and socio-economic factors. *Public Health Nutrition*.

[29] Abiba A., Grace A. N. K., Kubreziga K. C. (2012). Effects of dietary patterns on the nutritional status of upper primary school children in Tamale metropolis. *Pakistan Journal of Nutrition*.

[30] Wassie M. M., Gete A. A., Yesuf M. E., Alene G. D., Belay A., Moges T. (2015). Predictors of nutritional status of Ethiopian adolescent girls: a community based cross sectional study. *BMC Nutrition*.

[31] Kassa Tekile A., Abate Woya A., Basha G. W. (2019). Prevalence of malnutrition and associated factors among under-five children in Ethiopia: evidence from the 2016 Ethiopia Demographic and Health Survey. *BMC Research Notes*.

[32] Kt R., M A., Wakayo T. (2016). Nutritional status and its associated factors among school adolescent girls in Adama City, Central Ethiopia. *Journal of Nutrition & Food Sciences*.

